# Supplementary material for: Genome analysis of the monoclonal marbled crayfish reveals genetic separation over a short evolutionary timescale
Source: Commun Biol. 2021 Jan 18;4:74. doi: 10.1038/s42003-020-01588-8 (PMC7814009; doi:10.1038/s42003-020-01588-8)
Supplement: Supplementary file 2 — Supplementary Information [file 42003_2020_1588_MOESM2_ESM.pdf]

Supplementary Information

Genome analysis of the monoclonal marbled crayfish reveals genetic separation over a short evolutionary timescale

Contents:

Supplementary Figures S1-S3

Supplementary Tables S1-S5

Supplementary References

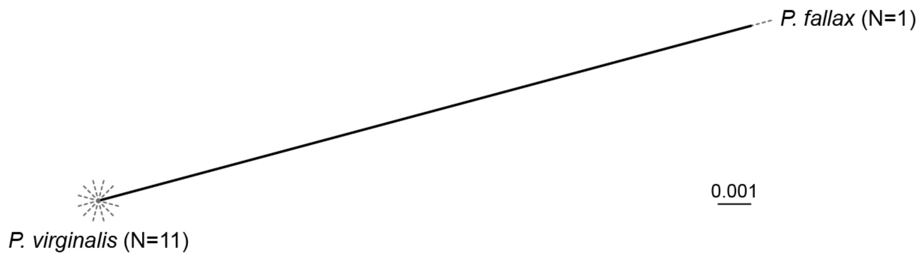

Fig. S1. Genetic relationships of mitochondrial genomes from the 11 marbled crayfish shown in Fig. 2A and the *P. fallax* mitochondrial genome reference sequence. The 11 independent marbled crayfish (*P. virginalis*) mitochondrial genome sequences were found to be identical and therefore form a single dot. The bar indicates the genetic distance.

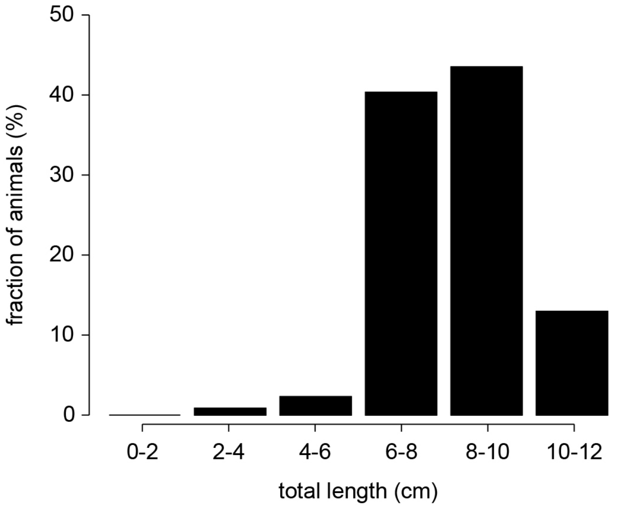

Fig. S2. Catch size distribution of the mark-recapture survey.

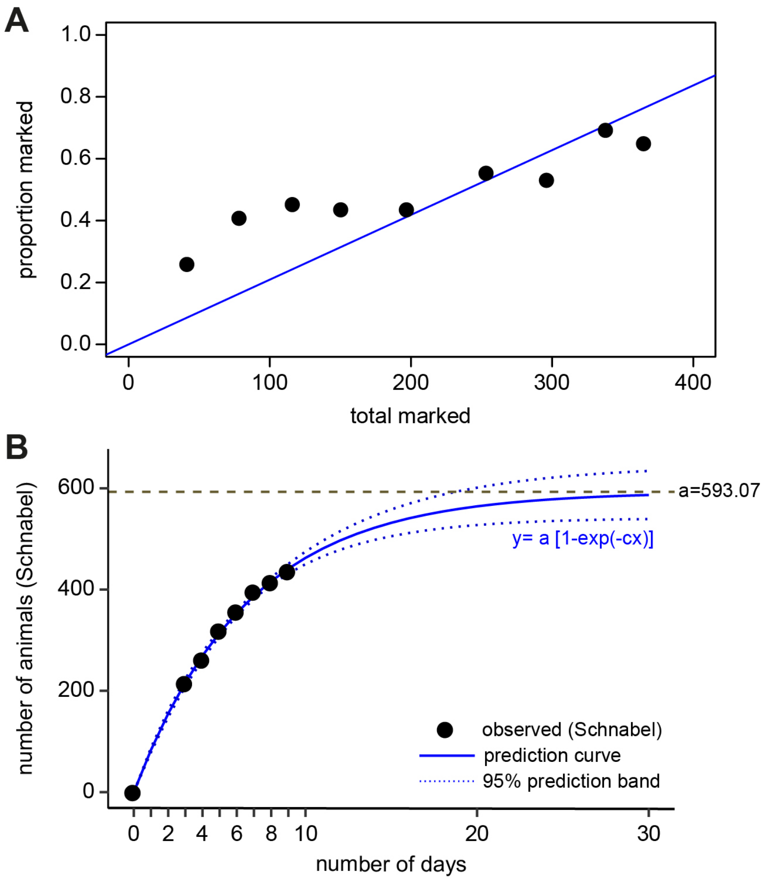

Fig. S3. Processing of mark-recapture data. (A) Relationship between the proportion of marked animals and the total of marked animals. A linear regression (blue line) has an adjusted R-square of 0.9171 (p=8.312e-06), indicating that the Schnabel model is valid. (B) Prediction curve of the number of animals (after application of the Schnabel formula) from a non-linear growth model.

Tab. S1. European marbled crayfish populations analyzed in this study.

#	Country	Population name	Coordinates	Source	Method	SRA accession no.
1	Germany	Reilingen	49.296893N, 8.544591E	ref. 1	WGS	SRS5949840
2		Singliser See	51.059816N, 9.305118E	S.T., 2017	WGS	SRS5949841
3		Moosweiher	48.030637N, 7.804174E	ref. 2	WGS	SRS5949846
4		Baggersee Epple	48.715240N, 9.563124E	(S.T., 2017)	PCR (4)	SRS7601340
5		Krumme Lanke	52.452560N, 13.233083E	(S.T., 2017)	PCR (4)	SRS7601480
6	Austria	Karlsbader Weiher	47.838388N, 13.012426E	(Daniela Latzer, 2019)	PCR (4)	SRS7592189
7	Czech Republic	Vodňany*	49.154157N, 14.169428E	ref. 3	WGS	SRS5949849
8	Slovakia	Bojnice	48.767190N, 18.577537E	(A.K., 2018)	WGS	SRS5949847
9		Leopoldov	48.453243N, 17.785830E	(A.K., 2018)	PCR (3)	SRS7601552
10		Bratislava	48.099839N, 17.129904E	(A.K., 2018)	PCR (3)	SRS7593237
11	Hungary	Danube	47.515540N, 19.042736E	(A.W., 2018)	WGS	SRS5949850
12	Romania	Băile Felix	46.988917N, 21.978694E	ref. 4	WGS	SRS5949851
13	Ukraine	Dnipro	48.505319N, 35.102377E	(R.N., 2018)	WGS	SRS5949852
14	Estonia	Narva	59.314708N, 28.084255E	ref. 5	WGS	SRS5949853
15	Malta	Ghajn il-Papri	36.022760N, 14.287933E	ref. 6	WGS	SRS5949848

All populations were genetically authenticated by whole-genome sequencing (WGS) or by PCR testing of multiple animals, as indicated in brackets. All animals that were tested had 0 polymorphisms in the mitochondrial cytochrome B marker and the nuclear Dnmt1 marker, as described in ref. 1. *Laboratory stock established from the likely source of populations in the Czech Republic.

Tab. S2. Whole-genome sequencing datasets.

Population name	Population # (from Tab. S1)	Number of read pairs	Mapping ratio (%)	Coverage
Reilingen, Germany	1	362,022,733	84.0	42.7X
Reilingen (2)	1	452,534,084	81.5	40.6X
Reilingen (3)	1	454,991,136	81.7	40.9X
Reilingen (4)	1	451,122,510	81.3	40.7X
Reilingen (5)	1	458,780,022	80.8	42.1X
Singliser See, Germany	2	431,347,724	82.0	39.6X
Moosweiher, Germany	3	420,501,584	82.9	40.2X
Czech Republic	7	425,171,972	81.6	39.0X
Slovakia	8	410,365,132	76.4	35.3X
Hungary	11	408,137,880	77.2	36.4X
Romania	12	446,242,415	81.7	41.5X
Ukraine	13	419,042,145	81.8	38.3X
Estonia	14	417,935,760	81.8	39.6X
Malta	15	412,900,336	82.7	38.6X
Ihosy, Madagascar	n.a. (from ref. 7)	452,157,698	80.3	36.8X

n.a.: not applicable

Tab. S3. Genetic variant effect types.

Type (alphabetical order)	Count	Fraction
3‘_UTR_variant	34	0.17%
5_prime_UTR_premature_start_codon_gain_variant	6	0.03%
5_prime_UTR_variant	18	0.09%
downstream_gene_variant	1,851	9.48%
intergenic_region	14,587	74.75%
intron_variant	844	4.32%
missense_variant	227	1.16%
missense_variant+splice_region_variant	2	0.01%
splice_acceptor_variant+intron_variant	1	0.005%
splice_region_variant	9	0.05%
splice_region_variant+intron_variant	1	0.005%
start_lost	2	0 %
stop_gained	5	0.03%
stop_lost+splice_region_variant	2	0.01%
synonymous_variant	168	0.86%
upstream_gene_variant	1,754	8.99%

Tab. S4. Base changes (SNVs).

	**A**	**C**	**G**	**T**
**A**	-	740	2,010	1,382
**C**	918	-	588	2,705
**G**	2,684	595	-	916
**T**	1,313	2,029	764	-

Tab. S5. Trapping of marbled crayfish at Lake Reilingen.

Trap no.	Trap depth	No. of animals
1	<2 m	77
2	<2 m	43
3	<2 m	35
4	<2 m	8
5	2-4 m	32
6	2-4 m	27
7	2-4 m	29
8	2-4 m	24
9	2-4 m	25
10	2-4 m	26
11	4-7 m	6
12	4-7 m	6
13	7-10 m	9
14	7-10 m	31
15	>10 m	16
16	>10 m	0
17	>10 m	0
18	>10 m	0

No. of animals represents the cumulative number of marked animals after 10 days.

Supplementary references

1. Gutekunst J., et al. Clonal genome evolution and rapid invasive spread of the marbled crayfish. *Nat. Ecol. Evol.* **2**, 567-573 (2018).

2. Vogt G., et al. The marbled crayfish as a paradigm for saltational speciation by autopolyploidy and parthenogenesis in animals. *Biol. Open* **4**, 1583-1594 (2015).

3. Patoka J., et al. Predictions of marbled crayfish establishment in conurbations fulfilled: Evidences from the Czech Republic. *Biologia* **71**, 1380-1385 (2016).

4. Pârvulescu L., Togor A., Lele S. F., Scheu S., Șinca D., Panteleit J. First established population of marbled crayfish Procambarus fallax (Hagen, 1870) f. virginalis (Decapoda, Cambaridae) in Romania. *Bioinvasions Rec.* **6**, 357-362 (2017).

5. Ercoli F., Kaldre K., Paaver T., Gross R. First record of an established marbled crayfish Procambarus virginalis (Lyko, 2017) population in Estonia. *Bioinvasions Rec.* **8**, 675-683 (2019).

6. Deidun A., et al. Invasion by non-indigenous freshwater decapods of Malta and Sicily, central Mediterranean Sea. *J. Crust. Biol.* **38**, 748-753 (2018).

7. Andriantsoa R., et al. Ecological plasticity and commercial impact of invasive marbled crayfish populations in Madagascar. *BMC Ecol.* **19**, 8 (2019).
