# Supplementary material for: Genome analysis of the monoclonal marbled crayfish reveals genetic separation over a short evolutionary timescale
Source: Commun Biol. 2021 Jan 18;4:74. doi: 10.1038/s42003-020-01588-8 (PMC7814009; doi:10.1038/s42003-020-01588-8)
Supplement: Supplementary file 3 — Reporting Summary [file 42003_2020_1588_MOESM3_ESM.pdf]

nature research

Corresponding author(s): Frank Lyko

Last updated by author(s): Oct 23, 2020

Reporting Summary

Nature Research wishes to improve the reproducibility of the work that we publish. This form provides structure for consistency and transparency in reporting. For further information on Nature Research policies, see Authors & Referees and the Editorial Policy Checklist .

Statistics

For all statistical analyses, confirm that the following items are present in the figure legend, table legend, main text, or Methods section.

n/a	Confirmed	
☐	☒	The exact sample size (*n*) for each experimental group/condition, given as a discrete number and unit of measurement
☒	☐	A statement on whether measurements were taken from distinct samples or whether the same sample was measured repeatedly
☒	☐	The statistical test(s) used AND whether they are one- or two-sided<br>*Only common tests should be described solely by name; describe more complex techniques in the Methods section.*
☒	☐	A description of all covariates tested
☒	☐	A description of any assumptions or corrections, such as tests of normality and adjustment for multiple comparisons
☐	☒	A full description of the statistical parameters including central tendency (e.g. means) or other basic estimates (e.g. regression coefficient) AND variation (e.g. standard deviation) or associated estimates of uncertainty (e.g. confidence intervals)
☐	☒	For null hypothesis testing, the test statistic (e.g. *F*, *t*, *r*) with confidence intervals, effect sizes, degrees of freedom and *P* value noted<br>*Give P values as exact values whenever suitable.*
☒	☐	For Bayesian analysis, information on the choice of priors and Markov chain Monte Carlo settings
☒	☐	For hierarchical and complex designs, identification of the appropriate level for tests and full reporting of outcomes
☒	☐	Estimates of effect sizes (e.g. Cohen's *d*, Pearson's *r*), indicating how they were calculated

Our web collection on statistics for biologists contains articles on many of the points above.

Software and code

Policy information about availability of computer code

Data collection	Quality control and trimming of WGS reads were controlled by FastQC and trimmomatic v.0.32
Data analysis	All software packages (including details, such as version numbers) and information for custom scripts are provided in the Methods and Code availability sections

For manuscripts utilizing custom algorithms or software that are central to the research but not yet described in published literature, software must be made available to editors/reviewers. We strongly encourage code deposition in a community repository (e.g. GitHub). See the Nature Research guidelines for submitting code & software for further information.

Data

Policy information about availability of data

All manuscripts must include a data availability statement. This statement should provide the following information, where applicable:

- Accession codes, unique identifiers, or web links for publicly available datasets
- A list of figures that have associated raw data
- A description of any restrictions on data availability

All sequencing data have been deposited as a NCBI BioProject (accession number PRJNA599283).

Field-specific reporting

Please select the one below that is the best fit for your research. If you are not sure, read the appropriate sections before making your selection.

Ecological, evolutionary & environmental sciences study design

All studies must disclose on these points even when the disclosure is negative.

Study description	Our study provides an approach for the challenging analysis of monoclonal genomes in asexual arthropods and uncovers evolutionary dynamics in the marbled crayfish genome over a very short evolutionary timescale. In addition, our findings also identify the rapid growth of marbled crayfish populations as an important factor for ecological monitoring.
Research sample	Fourteen marbled crayfish animals representing ten stable populations in Europe were sampled and a published dataset for four populations from Madagascar (Gutekunst et al, 2018) were used for our analysis.
Sampling strategy	Single representative animals for each population were collected without selection, while five animals were collected from the type locality
Data collection	Samples were collected by hand-catch from the shore.
Timing and spatial scale	Samples were collected during 2017-2019.
Data exclusions	No data has been excluded.
Reproducibility	WGS was performed once per each sample
Randomization	Samples from populations were taken without specific selection and single representative individuals were randomly chosen from the listed populations.
Blinding	Persons performing sample preparation and WGS were unaware of the sample identity.

Did the study involve field work? ☒ Yes ☐ No

Field work, collection and transport

Field conditions	As this is a genomic study, field conditions are not relevant.
Location	Provided in the Supplementary Table S1
Access and import/export	Public lakes, accessed by walking
Disturbance	There were no disturbances. The animals are considered as an invasive pest and were caught by hand-catch from the shore.

Reporting for specific materials, systems and methods

We require information from authors about some types of materials, experimental systems and methods used in many studies. Here, indicate whether each material, system or method listed is relevant to your study. If you are not sure if a list item applies to your research, read the appropriate section before selecting a response.

Materials & experimental systems

n/a	Involved in the study	
☒	☐	Antibodies
☒	☐	Eukaryotic cell lines
☒	☐	Palaeontology
☐	☒	Animals and other organisms
☒	☐	Human research participants
☒	☐	Clinical data

Methods

n/a	Involved in the study	
☒	☐	ChIP-seq
☒	☐	Flow cytometry
☒	☐	MRI-based neuroimaging

Animals and other organisms

Policy information about studies involving animals; ARRIVE guidelines recommended for reporting animal research

Laboratory animals	Not applicable
Wild animals	Procambarus virginalis (all-female). Animals were caught by hand and killed by decapitation immediately after catching.
Field-collected samples	Not applicable

Ethics oversight	Ethical approval was not required as the animals are considered an invasive pest.

Note that full information on the approval of the study protocol must also be provided in the manuscript.
